# Late follicular phase progesterone levels and *in vitro* fertilization and intracytoplasmic sperm injection outcomes

**DOI:** 10.3389/fendo.2026.1899934

**Published:** 2026-08-07

**Authors:** Hongfa Peng, Jingjing Jiang, Kaixuan Guo, Lei Jiang, Zechen Zhang, Jiahua Zheng, Yang Yang, Yuanyuan Peng, Wei Wang

**Affiliations:** 1 Department of Obstetrics and Gynecology, Second Hospital of Hebei Medical University, Shijiazhuang, China; 2 Department of Obstetrics and Gynecology, Hebei General Hospital, Shijiazhuang, China; 3 Department of Reproductive and Medicine, Second Hospital of Hebei Medical University, Shijiazhuang, China; 4 Department of Statistics, School of Public Health, Hebei Medical University, Shijiazhuang, China

**Keywords:** age, fresh embryo transfer, individualized medicine, IVF, live birth rate, ovarian stimulation, progesterone

## Abstract

**Objective:**

To study whether the impact of serum progesterone level on the day of hCG administration on reproductive outcomes varies across age groups and ovarian stimulation protocols in fresh in fresh *in vitro* fertilization (IVF) and intracytoplasmic sperm injection (ICSI) cycles.

**Methods:**

This single-center, retrospective cohort study was conducted from January 2014 to December 2024 and included 24,868 women undergoing their first fresh embryo transfer following an IVF or ICSI cycle at a university-affiliated fertility center. The exposure was serum progesterone level measured on the day of hCG administration. Primary outcomes were clinical pregnancy rate (CPR), miscarriage rate (MR) and live birth rate (LBR).

**Results:**

In women aged <35 years, progesterone was inversely associated with CPR (fully adjusted aOR=0.868, 95% CI 0.812–0.927, P<0.001) but positively associated with LBR after adjustment (aOR=1.090, 95% CI 1.019–1.167, P = 0.013). No significant effects were observed in patients aged ≥35 years. The progesterone–outcome relationship was significantly modified by protocol, with a nonlinear inverted U-shaped curve in GnRH-agonist protocols (interaction P<0.001 for CPR, P = 0.005 for LBR). In protocol-stratified analyses, progesterone was independently associated with reduced CPR in ultra-long GnRH-agonist (aOR=0.814, 95% CI 0.718–0.922, P = 0.001) and GnRH-antagonist protocols (aOR=0.849, 95% CI 0.771–0.936, P = 0.001), but not in the long GnRH-agonist protocol. The effect on LBR was positive in the long GnRH-agonist protocol (aOR=1.105, 95% CI 1.013–1.205, P = 0.024) and negative in the GnRH-antagonist protocol (aOR=0.884, 95% CI 0.788–0.991, P = 0.035). Sensitivity analyses confirmed the superiority of GnRH-agonist protocols across all progesterone levels (all adjusted P<0.01).

**Conclusion:**

The association between late follicular progesterone elevation and reproductive outcomes differs across ovarian stimulation protocols, demonstrating a nonlinear relationship with both clinical pregnancy and live birth. These results suggest that late follicular progesterone elevation should be interpreted with protocol-specific considerations.

## 1 Introduction

Progesterone elevation during the late follicular phase occurs in 12–38% of stimulated *in vitro* fertilization (IVF) cycles, despite gonadotropin-releasing hormone (GnRH) analog administration (1–3). Progesterone and estrogen jointly regulate endometrial development and receptivity, and progesterone supports early pregnancy. Previous studies have reported that elevated progesterone negatively affects outcomes in fresh IVF/intracytoplasmic sperm injection–embryo transfer (IVF/ICSI-ET) cycles (1, 4–6). Although a serum progesterone threshold of ≥ 1.5 ng/mL is widely accepted for predicting reduced pregnancy rates (1), age- and protocol-specific cutoffs remain undefined. This retrospective study aimed to determine age- and protocol-specific progesterone thresholds on the day of human chorionic gonadotropin (hCG) administration that are associated with optimal perinatal outcomes in fresh IVF/ICSI-ET cycles.

## 2 Materials and methods

### 2.1 Study design and patients

This single-center cohort study included women aged 20–45 years who underwent fresh IVF/ICSI-ET cycles at a hospital between January 2014 and December 2024. The study was conducted in accordance with the Declaration of Helsinki and approved by the Institutional Review Board of the Second Hospital of Hebei Medical University (2025-R791). The requirement for written informed consent was waived by the board due to the retrospective nature of the study. Exclusion criteria included chromosomal abnormalities in the partner, endometriosis, uterine pathologies (adenomyosis, submucosal fibroids, polyps, inflammation, or malformations), recurrent miscarriage, a history of reproductive surgery, a history of adverse pregnancy outcomes, and incomplete data.

### 2.2 Controlled ovarian stimulation protocols:

1. Long GnRH agonist (GnRH-a): 3.75 mg triptorelin acetate (Ferring GmbH, Ipsen Pharma) received on the mid-luteal phase or cycle day 1. After pituitary down-regulation (follicle ≤ 5 mm, estrogen < 50 pg/mL, luteinizing hormone [LH] < 5 mIU/mL), recombinant follicle-stimulating hormone (rFSH; Gonal-f, Merck Serono) was initiated at 100–300 IU/day.
2. Ultra-long GnRH-a: Monthly 3.75 mg triptorelin for 2–3 months; ovarian stimulation commenced 14 days after the last dose.
3. GnRH antagonist (GnRH-ant): rFSH started on cycle days 2–3. Cetrorelix/Orgalutran (0.25 mg/day) was added when the leading follicle reached 14 mm or after 6–7 days of rFSH. The initial gonadotrophin dose was individualized according to age, basal follicle-stimulating hormone (FSH) levels, antral follicle count, and body mass index (BMI). Dose adjustments were based on ovarian response, monitored by vaginal ultrasound and serum estradiol levels. Final oocyte maturation was triggered by hCG 36 hours before retrieval.

### 2.3 Embryo transfer and luteal support

Fresh cleavage-stage (day 3) or blastocyst-stage (day 5) transfers were performed under ultrasound guidance. Luteal phase support consisted of 90 mg vaginal progesterone gel (Crinone 8%; Merck) daily from oocyte retrieval until gestational sac visualization (5.5–6 weeks), and continued until 8 weeks post-transfer if pregnancy was achieved.

### 2.4 Data collection and outcomes

Demographic characteristics, laboratory parameters, and pregnancy outcomes were obtained from the institutional database.

#### 2.4.1 Primary outcomes

1. Clinical pregnancy rate (CPR): Presence of gestational sac(s) on ultrasound or histologically confirmed conception products.
2. Live birth rate (LBR): Delivery of live neonate(s) per transfer.
3. Miscarriage rate (MR): Pregnancy loss before 20 weeks (early: < 12 weeks; late: 12–20 weeks).

### 2.5 Statistical analysis

All analyses were stratified by age (<35, 35–<40, and 40–45 years) and ovarian stimulation protocol (long GnRH-a, ultra-long GnRH-a, and GnRH-ant). Serum progesterone levels on the day of hCG administration were analyzed both as a continuous variable and grouped into clinically defined strata. Associations between progesterone and clinical outcomes (CPR, MR and LBR) were estimated using logistic regression. To separate the direct effect of progesterone from confounding by ovarian response, a sequential adjustment strategy was employed: Model 1 adjusted for basic demographic and clinical covariates; Model 2 additionally adjusted for the number of oocytes retrieved; and Model 3 further adjusted for the number of top-quality embryos. This approach was applied consistently across age-stratified, protocol-stratified, and pooled analyses. Restricted cubic spline models with knots at the 5th, 35th, 65th, and 95th percentiles were fitted to examine potential nonlinear associations and to test interactions between progesterone and both age and stimulation protocol. Overall and nonlinear interaction terms were assessed using likelihood ratio tests. Model performance was evaluated by discrimination (area under the receiver operating characteristic curve) and calibration slope, with internal validation performed via bootstrap resampling to obtain optimism-corrected estimates. Multiplicative interactions between progesterone and both oocyte yield and anti-Müllerian hormone (AMH) were formally tested, and subgroup analyses stratified by ovarian response category were conducted. Independent predictors of CPR and LBR were identified using multivariable logistic regression. All tests were two-sided. P-values were adjusted for multiple comparisons using the Holm–Bonferroni method where applicable, and P < 0.05 was considered statistically significant. Analyses were performed using SPSS 26.0 (IBM Corp.) and R version 4.5.3.

## 3 Results

### 3.1 Patient baseline characteristics and treatment cycle overview

A total of 24,868 patients undergoing fresh IVF/ICSI-ET cycles were included in this study. Detailed patient characteristics and cycle outcomes are presented in **Supplementary Table 1**.

### 3.2 Results of group comparisons

#### 3.2.1 Baseline characteristics and progesterone effects stratified by age

Significant age-dependent differences were observed across all key baseline parameters (**Supplementary Table 2**; all P < 0.001), with the exception of basal FSH.

##### 3.2.1.1 Progesterone effects in patients aged < 35 years

In patients aged < 35 years, serum progesterone on the day of hCG administration, when analyzed as a continuous variable, was inversely associated with CPR even in the crude model (odds ratio [OR] = 0.912, 95% confidence interval [CI]: 0.860–0.967, *P* = 0.002; **Table 1**). This finding contrasts with the categorical analysis, in which the highest CPR and LBR were observed in the uppermost progesterone stratum (e.g., LBR 70.0% in the >2.5 ng/mL group), and both overall MR and early MR reached their lowest values in the 1.75–2.0 ng/mL interval (4.9% and 2.6%, respectively; **Table 2**). This discrepancy is attributable to confounding by ovarian response and embryo quality. After sequential adjustment for these factors, the inverse association between progesterone and CPR became more pronounced, with the fully adjusted OR reaching 0.868 (95% CI 0.812–0.927, *P* < 0.001; **Table 1**, Model 3).

**Table 1:** Sequential multivariable logistic regression analysis of the association between serum progesterone and clinical pregnancy rate in young patients (<35 years).

Model	Covariates	aOR	95% CI	P
Crude model	Unadjusted	0.912	(0.860, 0.967)	0.002
Model 1	Age, BMI, AMH, FSH, E2 and LH on hCG day	0.915	(0.859, 0.975)	0.006
Model 2	Model 1 + number of oocytes retrieved	0.864	(0.809, 0.923)	< 0.001
Model 3	Model 2 + embryo quality	0.868	(0.812, 0.927)	< 0.001

aOR, adjusted odds ratio; CI, confidence interval; BMI, body mass index; AMH, anti-Müllerian hormone; FSH, follicle-stimulating hormone; hCG, human chorionic gonadotropin; E2, estradiol; LH, luteinizing hormone.

**Table 2:** Impact of progesterone levels on clinical pregnancy rate (CPR), miscarriage rate (MR), and live birth rate (LBR) across different age groups.

Outcome	Age group	Progesterone level								Statistical test	
		p<1 ng/ml	p=1.0-1.25 ng/ml	p=1.25-1.5 ng/ml	p=1.5-1.75ng/ml	p=1.75-2.0ng/ml	p=2.0-2.25 ng/ml	p=2.25-2.5 ng/ml	p>2.5 ng/ml	χ²	p-value
CRP											
	<35 y	5824/8413(67.1%)	2341/3774(62.03%)	1873/2949(63.5%)	1219/1846(66.0%)	1425/2210(64.5%)	201/323(62.2%)	106/175(60.6%)	212/280(75.7%)	92.099	<0.001
	35-40 y	1010/2047(49.3%)	329/661(49.8%)	255/507(50.3%)	147/291(50.3%)	165/310(53.2%)	20/36(55.6%)	4/13(30.8%)	16/27(59.3%)	5.002	0.660
	40-45 y	154/623(24.72%)	52/163(31.9%)	24/94(25.53%)	12/54(22.22%)	17/56(30.36%)	4/9(44.44%)	0/2(0.0%)	1/5(20%)	6.726	0.454
MR											
	<35 y	484/5824(8.3%)a	205/2341(8.8%)	152/1873(8.1%)	78/1219(6.4%)	70/1425(4.9%)	8/201(4.0%)	6/106(5.7%)	15/212(7.1%)	29.331	<0.001
	35-40 y	175/1010(17.3%)	56/329(17.0%)	43/255(16.9%)	19/147(12.9%)	23/165(13.9%)	2/20(10.0%)	0/4(0.0%)	0/16(0.0%)	7.326	0.396
	40-45 y	47/154(30.5%)	16/52(30.8%)	8/24(33.3%)	3/12(25.0%)	5/17(29.4%)	1/4(25.0%)	0/2(0.0%)	0/1(0.0%)	1.637	0.977
EMR											
	<35 y	282/5824(4.8%)	133/2341(5.7%)	105/1873(5.6%)	52/1219(4.3%)	37/1425(2.6%)	4/201(2.0%)	3/106(2.8%)	7/212(3.3%)	28.245	<0.001
	35-40 y	122/1010(12.1%)	47/329(14.3%)	29/255(11.4%)	11/147(7.5%)	17/165(10.3%)	2/20(10.0%)	0/4(0.0%)	0/16(0.0%)	7.834	0.347
	40-45 y	43/154(27.9%)	13/52(25.0%)	5/24(20.8%)	3/12(25.0%)	5/17(29.4%)	1/4(25.0%)	0/2(0.0%)	0/1(0.0%)	1.793	0.970
LMR											
	<35 y	202/5824(3.5%)	72/2341(3.1%)	47/1873(2.5%)	26/1219(2.1%)	33/1425(2.3%)	4/201(2.0%)	3/106(2.83%)	8/212(3.77%)	12.609	0.082
	35-40 y	53/1010(5.2%)	9/329(2.7%)	14/255(5.5%)	8/147(5.4%)	6/165(3.6%)	0/20(0.0%)	0/4(0.0%)	0/16(0.0%)	6.511	0.482
	40-45 y	4/199(2.0%)	3/52(5.8%)	3/24(12.5%)	0/12(0.0%)	0/17(0.0%)	0/4(0.0%)	0/2(0.0%)	0/1(0.0%)	9.863	0.196
LBR											
	<35 y	4370/8413(51.9%)	2051/3774(54.3%)	1662/2949(56.4%)	1107/1846(60.0%)	1332/2210(60.3%)	188/323(58.2%)	98/175(56.0%)	196/280(70.0%)	138.545	<0.001
	35-40 y	756/2047(36.9%)	251/661(38.0%)	196/507(38.7%)	122/291(41.9%)	137/310(44.2%)	16/36(44.4%)	4/13(30.8%)	16/27(59.3%)	13.674	0.057
	40-45 y	94/623(15.1%)	36/163(22.1%)	16/94(17.0%)	7/54(13.0%)	12/56(21.4%)	3/12(25.0%)	0/2(0.0%)	1/5(20.0%)	7.027	0.426

Clinical pregnancy rate: CPR; Miscarriage rate: MR; Early miscarriage rate: EMR; Late miscarriage rate: LMR; Live birth rate (LBR).

The association between progesterone and LBR followed a distinctly different pattern. No significant association was observed in the crude model (OR = 1.014, 95% CI 0.956–1.074, *P* = 0.650). However, after sequential adjustment for covariates, oocyte yield, and embryo quality, a modest but significant positive association emerged. In the fully adjusted model, higher progesterone was independently associated with increased odds of live birth (aOR = 1.090, 95% CI 1.019–1.167, *P* = 0.013; **Table 1**, Model 3).

##### 3.2.1.2 Progesterone effects in patients aged ≥ 35 years

In patients aged ≥ 35 years, no significant progesterone-dependent effects on pregnancy outcomes were detected in either categorical or continuous analyses. Neither the crude nor the fully adjusted models revealed a statistically significant association between progesterone and CPR or LBR. Compared with patients aged < 35 years, CPR was significantly lower in those aged > 40 years (OR = 0.213, 95% CI: 0.184–0.246, *P* < 0.001; **Supplementary Table 3**).

##### 3.2.1.3 Determinants of CPR in younger patients

To explore factors underlying the variation in CPR across progesterone subgroups in the < 35 years cohort, baseline and ovarian stimulation characteristics were compared (**Supplementary Table 4**). Progesterone levels correlated inversely with BMI and positively with AMH, estradiol on hCG day, and total retrieved oocytes (all P < 0.001). Multivariate logistic regression identified age, BMI, stimulation protocol, and serum progesterone, estradiol, and LH levels on the day of hCG as independent predictors of CPR (**Supplementary Table 5**). Notably, in this multivariate model, which did not adjust for oocyte yield or embryo quality, progesterone remained an independent negative predictor, consistent with the continuous analyses presented in **Table 1**.

#### 3.2.2 Ovarian stimulation protocol and progesterone effects

Baseline characteristics differed significantly across protocols (**Supplementary Table 6**; *P* < 0.001). Patients treated with the GnRH-ant protocol were older and had lower AMH compared with those receiving either GnRH-a protocol. Estradiol on the day of hCG and oocyte yield were progressively higher in the GnRH-agonist-based regimens.

##### 3.2.2.1 Progesterone effects within protocol groups

Progesterone levels were significantly associated with CPR, MR, and LBR across all protocols, but the direction and confounding structure differed markedly by protocol (**Supplementary Table 7**; all *P* < 0.05). Sequential multivariable adjustment was essential to separate these effects from the confounding influence of ovarian response (**Table 3**).

**Table 3:** Independent effect of progesterone on clinical pregnancy rate, stratified by ovarian stimulation protocol: sequential adjustment models.

Protocol	Model	Adjustment variables	aOR (95% CI)	P
Long GnRH-a	Crude model	Unadjusted	0.916 (0.848, 0.989)	0.024
	Model 1	Age, BMI, AMH, FSH, E2 and LH on hCG day	1.001 (0.903, 1.11)	0.983
	Model 2	Model 1 + number of oocytes retrieved	0.981 (0.881, 1.092)	0.730
	Model 3	Model 2 + embryo quality	0.984 (0.884, 1.096)	0.772
Ultra-long GnRH-a	Crude model	Unadjusted	0.891 (0.801, 0.991)	0.034
	Model 1	Age, BMI, AMH, FSH, E2 and LH on hCG day	0.874 (0.775, 0.987)	0.029
	Model 2	Model 1 + number of oocytes retrieved	0.811 (0.716, 0.919)	0.001
	Model 3	Model 2 + embryo quality	0.814 (0.718, 0.922)	0.001
GnRH-ant	Crude model	Unadjusted	1.085 (0.986, 1.194)	0.094
	Model 1	Age, BMI, AMH, FSH, E2 and LH on hCG day	1.236 (1.144, 1.335)	<0.001
	Model 2	Model 1 + number of oocytes retrieved	0.846 (0.767, 0.932)	0.001
	Model 3	Model 2 + embryo quality	0.849 (0.771, 0.936)	0.001

aOR, adjusted odds ratio; CI, confidence interval; BMI, body mass index; AMH, anti-Müllerian hormone; FSH, follicle-stimulating hormone; hCG, human chorionic gonadotropin; E2, estradiol; LH, luteinizing hormone.

In the long GnRH-a protoco, the crude analysis revealed a significant inverse association between progesterone and CPR (OR = 0.916, 95% CI 0.848–0.989, *P* = 0.024). This association was attenuated and no longer statistically significant after full adjustment for covariates, oocyte yield, and embryo quality (Model 3 aOR = 0.984, 95% CI 0.884–1.096, *P* = 0.772). In contrast, a significant positive association between progesterone and LBR emerged and persisted in the fully adjusted model (Model 3 aOR = 1.105, 95% CI 1.013–1.205, *P* = 0.024; **Table 3**).

In the ultra-long GnRH-a protocol, a consistent and significant inverse relationship between progesterone and CPR was observed across all models. The fully adjusted OR for clinical pregnancy was 0.814 (95% CI 0.718–0.922, *P* = 0.001; **Table 3**). The association with LBR followed a pattern similar to that observed in the long protocol, with a positive trend that approached, but did not reach, statistical significance in the fully adjusted model (Model 3 aOR = 1.115, 95% CI 0.980–1.270, *P* = 0.099).

In the GnRH-ant protocol, the crude model showed a non-significant positive association between progesterone and CPR (OR = 1.085, 95% CI 0.986–1.194, *P* = 0.094), which contrasted sharply with a significant negative crude association with LBR (OR = 0.791, 95% CI 0.710–0.882, *P* < 0.001). After adjustment for basic covariates, the association with CPR became strongly positive (Model 1 aOR = 1.236, *P* < 0.001), a hallmark of negative confounding. Critically, introducing the number of oocytes retrieved in Model 2 completely reversed this direction, yielding a significant negative association (aOR = 0.846, 95% CI 0.767–0.932, *P* = 0.001), which persisted in the fully adjusted model (Model 3 aOR = 0.849, 95% CI 0.771–0.936, *P* = 0.001; **Table 3**). The fully adjusted aOR for LBR was 0.884 (95% CI 0.788–0.991, *P* = 0.035). While these protocol-stratified analyses reveal distinct linear associations between progesterone and treatment outcomes, they do not formally test whether the shape of the progesterone–outcome relationship differs across protocols. Therefore, restricted cubic spline analyses with formal interaction testing were performed to characterize potential nonlinearities and protocol-specific dose–response curves.

##### 3.2.2.2 Nonlinear interactions between progesterone and stimulation protocol

**Figure 1:**
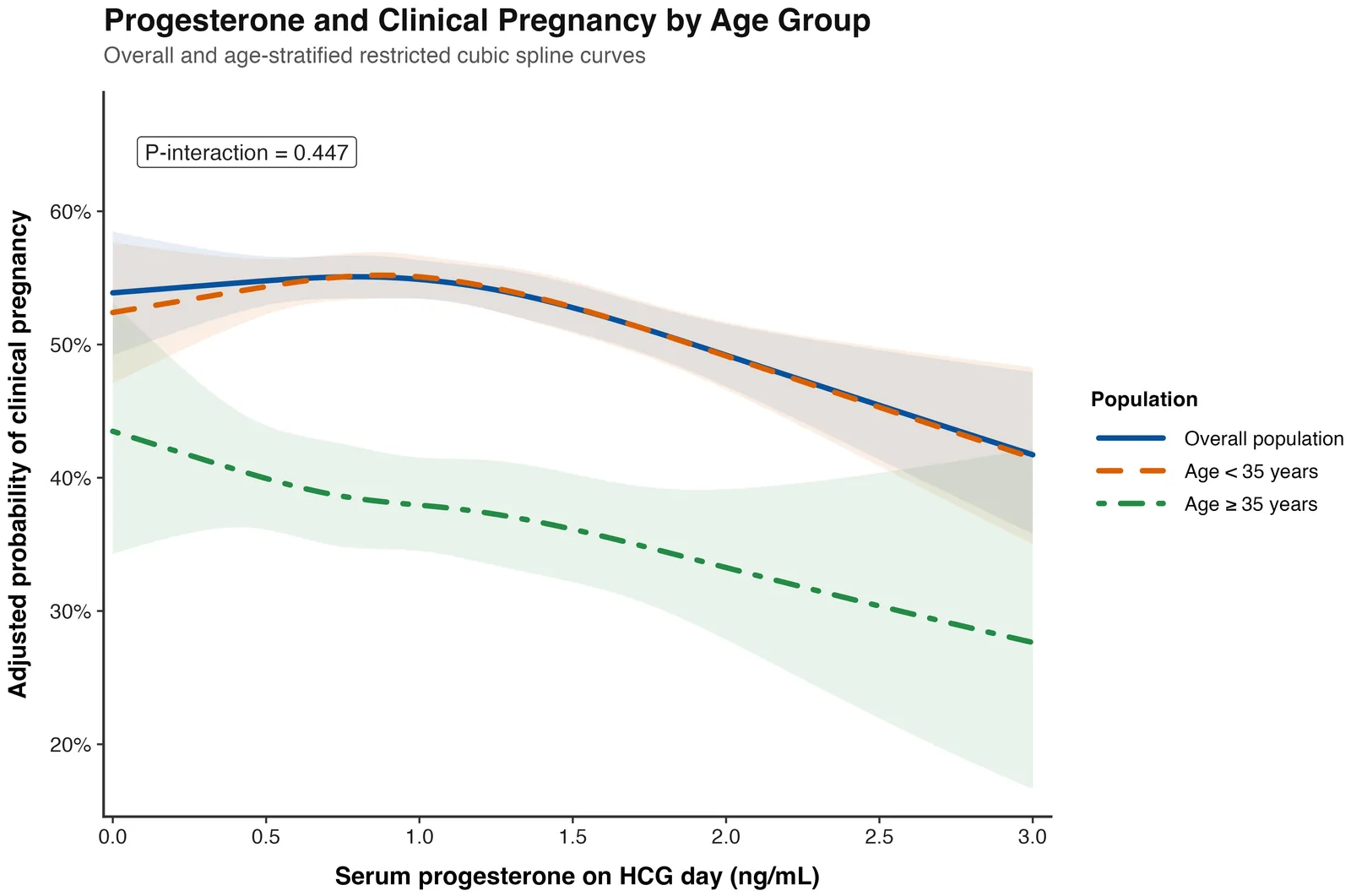
Multivariable-adjusted association between serum progesterone level on the day of HCG administration and clinical pregnancy rate, stratified by age group. Restricted cubic spline curves for the association between serum progesterone level on hCG day and adjusted probability of live birth, stratified by age groups. The solid lines represent the adjusted probabilities; shaded areas indicate 95% confidence intervals. Curves were derived from multivariable logistic regression models with a 4-knot restricted cubic spline for progesterone, adjusted for age (4-knot RCS), BMI, stimulation protocol, AMH, total gonadotropin dose, E2 on hCG day, FSH, oocytes retrieved, and total high-quality embryos. The *P* for interaction between progesterone and age group was 0.447, indicating no significant effect modification. Results were pooled across 50 multiple imputations.

**Figure 2:**
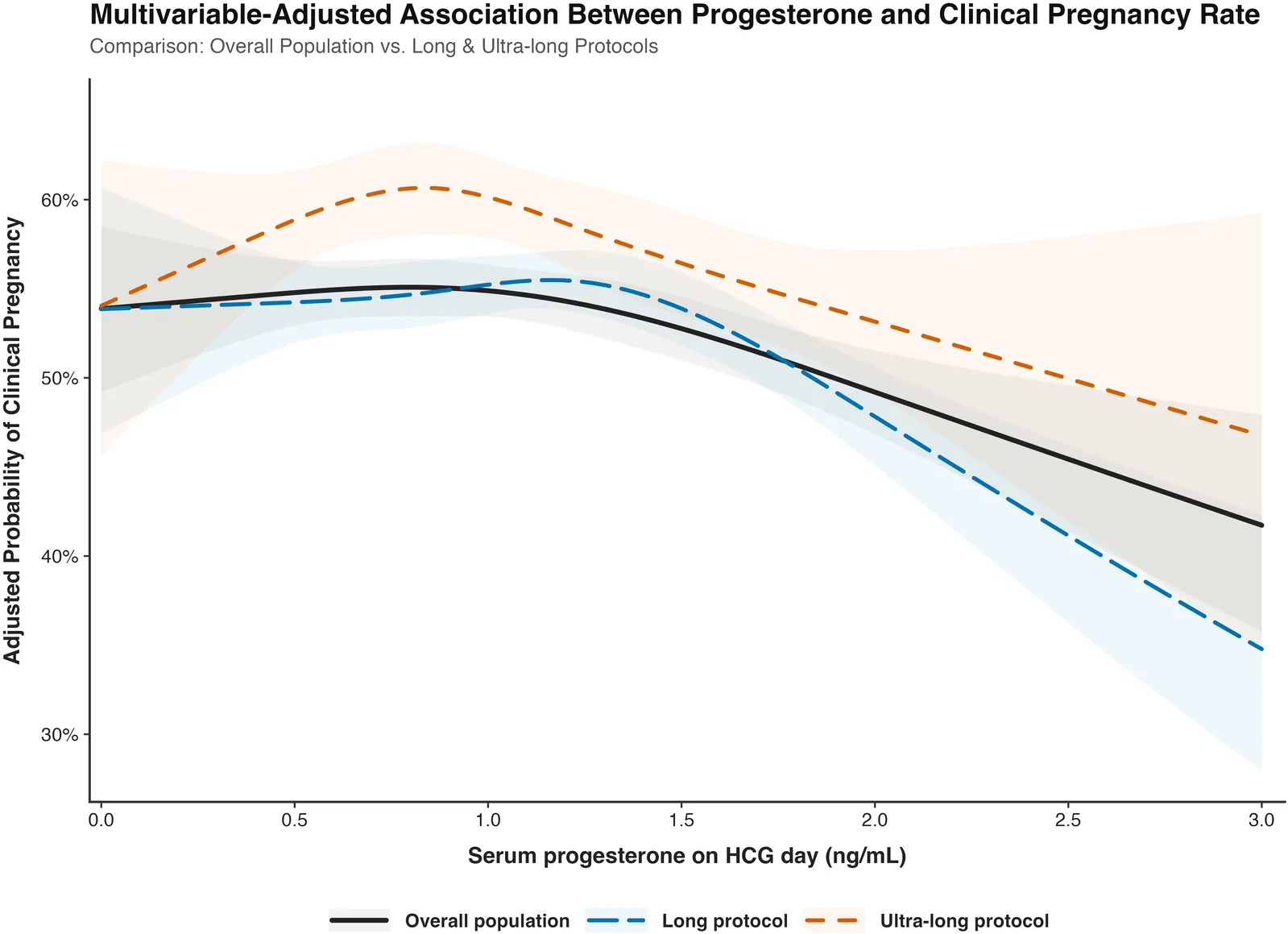
Multivariable-adjusted association between serum progesterone level on the day of HCG administration and clinical pregnancy rate, comparing the overall population with patients undergoing long and ultra-long protocols. Restricted cubic spline (RCS) models were used to flexibly model the relationship, with adjustments for multiple confounding variables. Solid lines represent the estimated probability of clinical pregnancy, and shaded areas indicate the 95% confidence intervals. The analysis was performed using 50 multiple imputations to handle missing data. The overall population is shown in blue, the long protocol in red, and the ultra-long protocol in green (colors to be specified based on your figure).

No significant interaction was detected between progesterone and age for either live birth ( $P_{interaction}$ = 0.447) (**Figure 1**) or clinical pregnancy ( $P_{interaction}$ = 0.680). In contrast, a significant nonlinear interaction between progesterone and stimulation protocol was observed for both outcomes (live birth: $P_{overall\;interaction}$ = 0.005, $P_{nonlinear}$ = 0.014; clinical pregnancy: $P_{overall\;interaction}$ < 0.001, $P_{nonlinear}$ = 0.022). As illustrated in **Figure 2**, in the long and ultra-long GnRH-a protocols, the predicted probability of clinical pregnancy showed an inverted U-shaped relationship with progesterone levels, rising initially before declining at higher concentrations. The curve for the GnRH-ant protocol remained comparatively flat, and the predicted probabilities were substantially lower than those for the GnRH-agonist-based protocols across the entire progesterone range.

**Figure 3:**
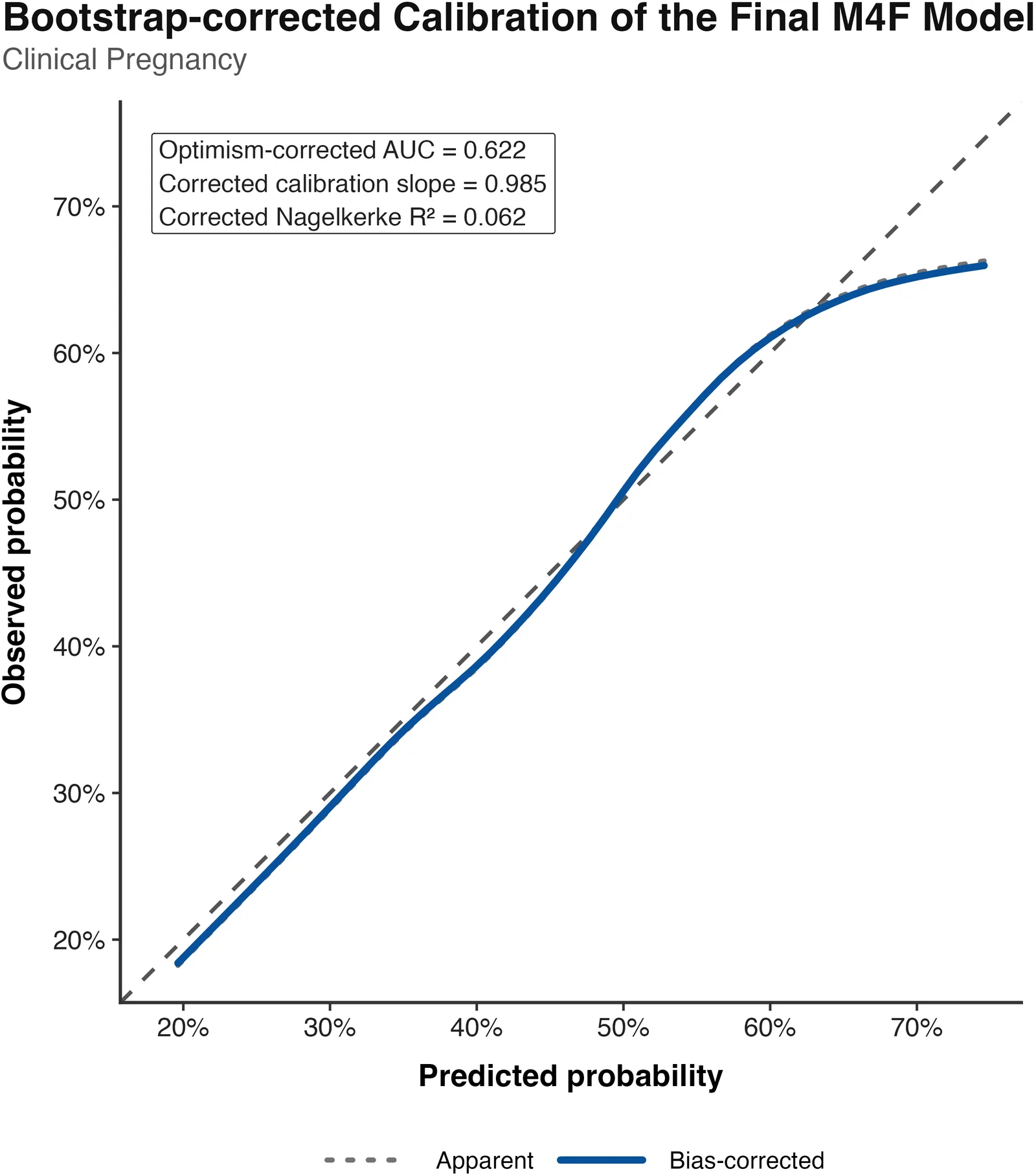
Calibration plot of the final multivariable model for predicting clinical pregnancy. Restricted cubic spline curve for the association between serum progesterone level on hCG day and adjusted probability of live birth in the ultra-long stimulation protocol. The solid line represents the adjusted probability; the shaded area indicates the 95% confidence interval. The curve was derived from a multivariable logistic regression model with a 4-knot restricted cubic spline for progesterone, adjusted for age (4-knot RCS), BMI, AMH, total gonadotropin dose, E2 on hCG day, FSH, oocytes retrieved, and total high-quality embryos. Total *N* = 6,347, with 3,779 live births. Results were pooled across 50 multiple imputations.

**Figure 4:**
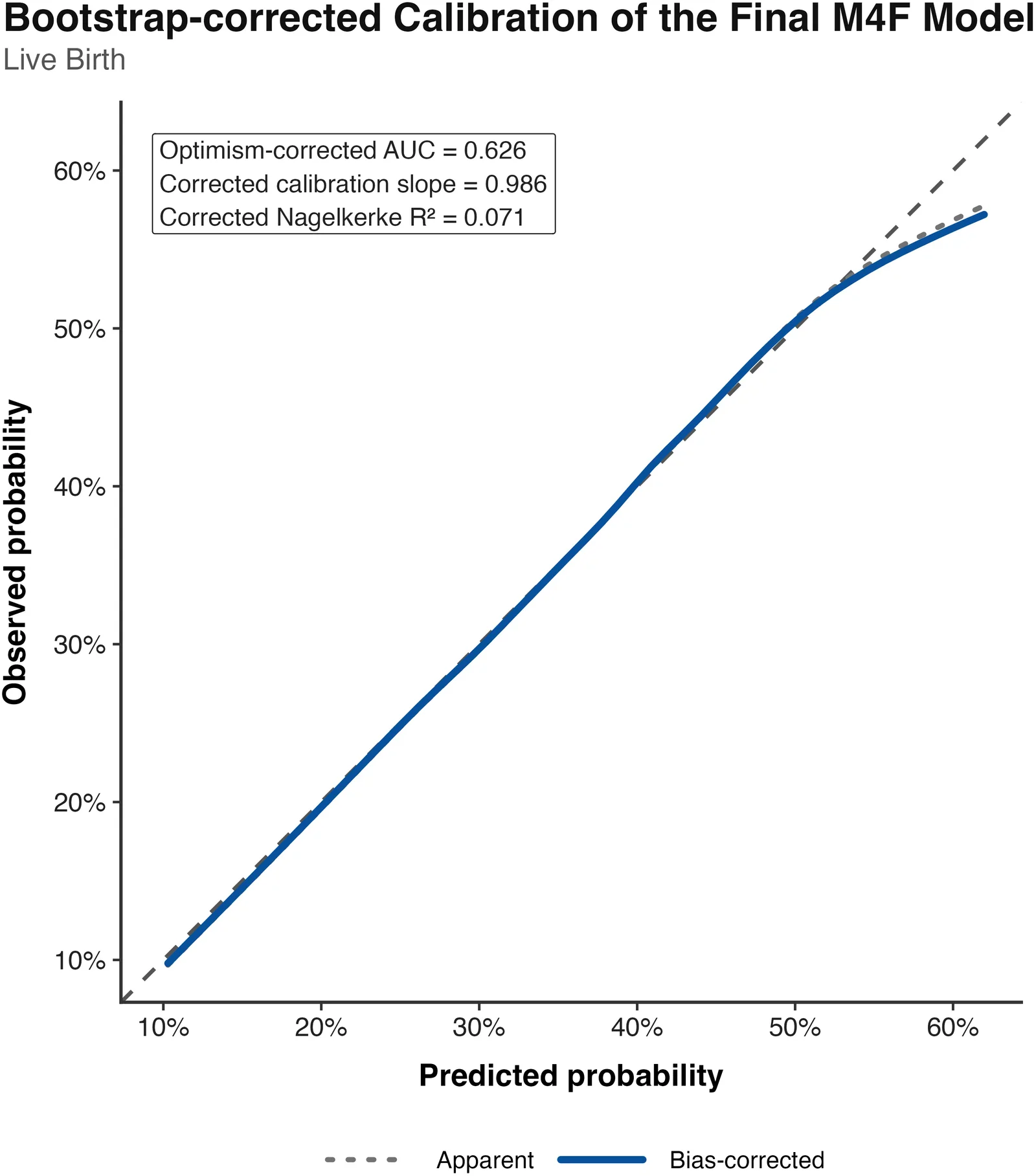
Calibration plot of the final multivariable model for predicting live birth. Calibration plot for the prediction model of live birth. The dashed diagonal line indicates perfect calibration. Observed live birth rates with 95% confidence intervals (error bars) are plotted against predicted probabilities across 6 risk groups (10%–60%). Calibration was assessed by the Hosmer-Lemeshow test (*P* < 0.01) and calibration slope (0.99). Live birth was defined as delivery of a viable infant beyond 28 weeks of gestation. The model was developed using binary logistic regression with live birth as the outcome (1 = live birth, 0 = no live birth). Total *N* = 24,868, with 13,266 live birth events.

Internal validation of the full prediction model incorporating these interactions demonstrated good calibration (corrected calibration slope ≈ 0.99 for both models) with minimal overfitting (decrease in AUC after optimism correction < 0.003). The discriminative performance was moderate (optimism-corrected AUC: 0.622 for clinical pregnancy; 0.626 for live birth) (**Figures 3**, **4**), and Nagelkerke R² indicated limited overall explanatory power. Sensitivity analysis further confirmed the robustness of the protocol effects: across the entire observed progesterone range, both the long and ultra-long GnRH-a protocols were associated with significantly higher adjusted odds of clinical pregnancy and live birth compared with the GnRH-ant protocol (all $P_{Holm}$ < 0.01), with no local intervals of non-significance detected.

#### 3.2.3 Overall progesterone effects in the full cohort

In the pooled analysis, higher progesterone levels were associated with younger age, lower BMI, and higher markers of ovarian reserve and response (**Supplementary Table 8**; all *P* < 0.001). Consequently, the group with progesterone > 2.5 ng/mL exhibited the highest crude CPR (73.4%), the lowest MR (4.8%), and the highest crude LBR (67.9%).

#### 3.2.4 Multivariable regression and causal pathway analysis

The strong confounding by ovarian response necessitated a rigorous causal pathway analysis. In a multivariable model that did not adjust for oocyte yield or embryo quality, progesterone level on the day of hCG was independently associated with a slightly increased CPR (aOR = 1.099, 95% CI 1.035–1.168, *P* = 0.002), but showed no significant association with LBR (*P* = 0.281). Age <35 years, GnRH-ant protocol, lower AMH levels, and IVF (vs. ICSI) were independent favorable factors for both CPR and LBR (all *P* < 0.05). Among these, stimulation protocol exerted the greatest effect on LBR (OR for long GnRH-a vs. GnRH-ant: 0.520, 95% CI 0.481–0.562), followed by age (OR for ≥ 35 vs. < 35 years: 0.559, 95% CI 0.516–0.606) (**Supplementary Table 5**).

To directly address the confounding by ovarian response, sequential logistic regression models that progressively accounted for oocyte yield and embryo quality were constructed. The adjusted ORs were 0.934 (95% CI 0.885–0.985) in Model 1, 0.875 (0.827–0.926) after further adjustment for oocyte number (Model 2), and 0.877 (0.829–0.929) in the fully adjusted Model 3 (all *P* < 0.05). This inverse independent effect was confirmed in the age-stratified analysis for patients aged < 35 years (**Table 1**) and across all stimulation protocols (**Table 3**).

Significant multiplicative interactions were detected between progesterone level and both oocyte yield (likelihood ratio χ² = 12.683, *P* for interaction < 0.001) and AMH (likelihood ratio χ² = 21.576, *P* for interaction < 0.001), confirming that the effect of progesterone is modified by ovarian response and reserve. Notably, this effect modification by ovarian response is distinct from the progesterone-by-protocol interaction reported in Section 2.2, which remained significant after adjustment for oocyte yield, indicating that stimulation protocol modifies the progesterone–outcome relationship through pathways that are at least partially independent of ovarian response. Accordingly, subgroup analyses stratified by oocyte yield were conducted. Progesterone was significantly associated with lower clinical pregnancy in the low-response (OR = 0.811, *P* = 0.006) and normal-response (OR = 0.900, *P* = 0.002) groups. Although the association did not reach statistical significance in the high-response group (OR = 0.872, *P* = 0.24), the point estimate remained consistently below 1, indicating no evidence of a protective effect even in this subgroup.

## 4 Discussion

This large retrospective study demonstrates a classic example of Simpson’s paradox in the relationship between serum progesterone on the day of hCG and reproductive outcomes in fresh IVF/ICSI cycles. In crude categorical analyses, elevated progesterone appeared to be associated with remarkably favorable outcomes—the highest CPR (73.4%), the lowest MR (4.8%), and the highest LBR (67.9%) were all observed in the > 2.5 ng/mL stratum. However, sequential multivariable adjustment for ovarian response and embryo quality systematically exposed an independent inverse association between progesterone and clinical pregnancy, while revealing a striking protocol-dependent divergence in its effect on live birth. These findings underscore the critical importance of accounting for the confounding architecture inherent in progesterone-outcome analyses and provide new insights into the effect modification exerted by age and stimulation protocol.

The discordance between crude and adjusted estimates observed in this study reflects a fundamental confounding structure that has likely contributed to the long-standing controversy surrounding progesterone thresholds in fresh IVF cycles (7–9). Patients with elevated progesterone on the day of hCG differed systematically from those with lower levels: they were younger, had lower BMI, higher AMH, produced significantly more oocytes, and had a greater number of top-quality embryos available for transfer (**Table 2**, **Supplementary Table 8**). Each of these factors is independently associated with higher pregnancy and live birth rates, creating a potent positive confounding effect that masks the underlying negative association between progesterone and clinical outcomes (10).

The sequential modeling approach employed in this study makes this confounding structure transparent. In the overall cohort, a multivariable model that did not adjust for oocyte yield or embryo quality still yielded a positive association between progesterone and clinical pregnancy (aOR = 1.099, *P* = 0.002). The critical reversal occurred with the introduction of the number of oocytes retrieved (aOR = 0.875), and the estimate remained stable after further adjustment for embryo quality (aOR = 0.877; **Table 3**). This pattern was replicated in the age-stratified analysis: in patients aged < 35 years, where crude categorical data gave the misleading impression of a linear increase in LBR with rising progesterone, the fully adjusted aOR for clinical pregnancy was 0.868 (*P* < 0.001). The reversal was particularly dramatic in the antagonist protocol, where the crude non-significant positive trend (OR = 1.085, *P* = 0.094) reversed to a significant negative association after adjustment for oocyte number (aOR = 0.846, *P* = 0.001) and remained negative in the fully adjusted model (aOR = 0.849, *P* = 0.001). The consistency of this reversal across sub-analyses identifies oocyte yield as the dominant confounder—a finding consistent with prior work demonstrating that the number of oocytes retrieved is the single most important variable mediating the relationship between progesterone and pregnancy outcomes (11, 12). An important methodological implication is that studies failing to adequately adjust for ovarian response—particularly the number of oocytes retrieved—are likely to produce biased estimates that systematically underestimate the detrimental effect of progesterone.

After full adjustment, elevated progesterone was independently associated with reduced CPRs across nearly all strata examined. In patients aged < 35 years, the fully adjusted aOR was 0.868 (*P* < 0.001). The negative association was most pronounced in the ultra-long GnRH-a protocol (aOR = 0.814, *P* = 0.001) and the GnRH-ant protocol (aOR = 0.849, *P* = 0.001). Notably, the ultra-long GnRH-a protocol was the only regimen in which the inverse association was already significant in the crude model (OR = 0.891, *P* = 0.034) and remained consistently significant across all sequential models, suggesting that the negative signal in this protocol is sufficiently strong to be detected even without adjustment. In the long GnRH-a protocol, the crude inverse association (OR = 0.916, *P* = 0.024) was progressively attenuated with the introduction of oocyte yield and embryo quality, becoming non-significant in the fully adjusted model (aOR = 0.984, *P* = 0.772). Even in this stratum, however, the point estimate remained below unity, consistent with a weak residual negative signal. In patients aged ≥ 35 years, no significant progesterone-dependent effects on either clinical pregnancy or live birth were detected, likely reflecting the overwhelming influence of age-related factors—a conclusion supported by the markedly lower CPR in patients aged > 40 years compared with those < 35 years (OR = 0.213, *P* < 0.001) and consistent with the well-established dominance of embryo aneuploidy in determining reproductive failure in older women (13).

The consistency of the inverse association with clinical pregnancy across diverse clinical contexts strongly supports a genuine biological effect of progesterone on the endometrium during the implantation window. The concept that supraphysiological progesterone impairs endometrial receptivity is biologically coherent. In natural cycles, progesterone rises only after the LH surge, initiating secretory transformation. Premature elevation during the late follicular phase of COS is therefore thought to advance the window of implantation, producing embryo–endometrial asynchrony and compromising implantation (8). Endometrial gene expression studies have demonstrated that progesterone elevation alters the transcription of genes involved in receptivity, including those regulating cell adhesion, immune modulation, and decidualization (14). Mechanistically, this has been linked to aberrant epigenetic modifications, such as altered histone marks, that dysregulate key receptivity genes like *HOXA10*, thereby disturbing the precise timing of the implantation window (15). The significant interactions between progesterone and both oocyte yield (*P* for interaction < 0.001) and AMH (*P* for interaction < 0.001) observed in the present study further suggest that endometrial susceptibility to progesterone may be modulated by the overall intensity of ovarian stimulation—a finding consistent with the hypothesis that a supraphysiologic hormonal milieu disrupts the endocrine dialogue at the maternal-fetal interface (9, 16).

A further novel finding of this study is the demonstration of a significant nonlinear interaction between progesterone and stimulation protocol for both clinical pregnancy and live birth. Restricted cubic spline analyses, adjusted for age, BMI, stimulation protocol, gonadotropin dose, estradiol and FSH on the day of hCG, number of oocytes retrieved, and number of top-quality embryos, confirmed that the shape of the progesterone–outcome relationship differs fundamentally across protocols. In the long and ultra-long GnRH-a protocols, the predicted probability of clinical pregnancy exhibited an inverted U-shaped relationship with progesterone, rising at moderate levels and declining at higher concentrations. In contrast, the curve for the GnRH-ant protocol remained comparatively flat, with substantially lower predicted probabilities across the entire progesterone range. This nonlinear pattern explains why the linear effect of progesterone on clinical pregnancy in the long GnRH-a protocol was not significant after full adjustment (aOR = 0.984, P = 0.772; Section 2.1): the opposing directions of the ascending and descending segments of the inverted U-shaped curve averaged toward the null in a linear model. The presence of an ascending limb in the agonist protocols suggests that moderate progesterone elevation—within a certain range—may reflect a more robust hormonal milieu without yet reaching the threshold at which endometrial advancement becomes detrimental. The absence of such an ascending limb in the antagonist protocol is consistent with a narrower therapeutic window, wherein even modest progesterone elevations may compromise receptivity. Sensitivity analysis further confirmed the robustness of these protocol-specific patterns: across the entire observed progesterone range, both the long and ultra-long GnRH-a protocols were associated with significantly higher adjusted odds of clinical pregnancy and live birth compared with the GnRH-ant protocol (all adjusted P < 0.01), with no local intervals of non-significance detected. This global superiority, verified by rigorous interval testing, strengthens the evidence base for differential clinical management by protocol type.

The most novel finding of this study is the marked divergence in the association between progesterone and live birth across stimulation protocols. This divergence carries important mechanistic and clinical implications, suggesting that the effect of progesterone is not confined to the implantation window but may, in certain protocols, extend to influence the probability of live birth even after clinical pregnancy has been established.

In the GnRH-ant protocol, progesterone was independently and significantly associated with reduced live birth both in the crude model (OR = 0.791, *P* < 0.001) and after full adjustment for all covariates including embryo quality (aOR = 0.884, *P* = 0.035). The fact that the negative association was evident even before adjustment indicates that the adverse signal was sufficiently strong that not even the substantial positive confounding by the larger number of oocytes and superior embryo quality in high-progesterone cycles could obscure it. The persistence of this negative association after full adjustment argues against the explanation that the lower LBR merely reflects poorer embryo quality. Although the present study cannot distinguish between implantation failure, biochemical pregnancy loss, and clinical miscarriage as the precise locus of the post-implantation effect, the data are compatible with a persistent detrimental influence of progesterone in antagonist cycles that extends beyond implantation to compromise the probability of live birth. Potential mechanisms include suboptimal decidualization, impaired trophoblast invasion, and dysregulated maternal-fetal immune crosstalk (17, 18). The absence of pituitary downregulation in antagonist cycles may render the endometrium more vulnerable to such perturbations, as it develops in a less controlled endocrine environment (19). Additionally, antagonist cycles may selectively enrich for patients with underlying endometrial pathology—such as endometriosis or adenomyosis—in whom progesterone sensitivity may be heightened; this remains an important area for future investigation.

In the long GnRH-a protocol, the association between progesterone and live birth showed a distinctly different pattern. The crude association was non-significant, but after full adjustment a significant positive association emerged (aOR = 1.105, *P* = 0.024). A similar pattern was observed in patients aged < 35 years in the overall cohort: the crude association was null (OR = 1.014, *P* = 0.650), becoming significantly positive after full adjustment (aOR = 1.090, *P* = 0.013). These two findings may partially overlap, as younger patients are more likely to have been treated with long agonist protocols; however, the consistency of the pattern across both age and protocol strata is noteworthy.

The sequential modeling data help explain this pattern. In the long agonist protocol, the crude inverse association between progesterone and clinical pregnancy (OR = 0.916, *P* = 0.024) was progressively attenuated as oocyte yield and embryo quality were introduced into the model, while the association with live birth simultaneously shifted from non-significant to significantly positive. This suggests a selection-based buffering mechanism: under conditions of a more synchronized endometrial environment—achieved either through pituitary downregulation or inherent to younger age—only embryos of sufficient developmental competence successfully implant. This interpretation aligns with evidence that GnRH-a protocols produce a more uniform and controlled endometrial milieu compared with antagonist protocols (20, 21). The elevated progesterone may then serve as a marker of a more robust luteal phase that supports ongoing pregnancy, a concept consistent with the physiological role of progesterone in early pregnancy maintenance (22). In this model, the embryos that survive the implantation hurdle are of higher quality, and the hormonal milieu that posed a barrier at implantation may become supportive thereafter.

The ultra-long GnRH-a protocol occupied an intermediate position. The fully adjusted association between progesterone and live birth showed a positive trend that approached but did not reach statistical significance (aOR = 1.115, *P* = 0.099). Coupled with its consistently significant negative association with clinical pregnancy across all models, this pattern suggests that in the ultra-long protocol the implantation-stage detriment is pronounced, while the post-implantation buffering effect is less robust than in the long agonist protocol. The prolonged pituitary suppression inherent to the ultra-long protocol may differentially affect endometrial receptivity and subsequent luteal phase function (23).

Although the retrospective design of this study precludes definitive clinical recommendations, the findings have implications for risk stratification in patients undergoing fresh embryo transfer. The results suggest that the interpretation of an elevated progesterone level should be informed not only by the progesterone value itself but also by the stimulation protocol employed and the patient’s ovarian response. In antagonist cycles, where the negative association with live birth persists after full adjustment and is not buffered by embryo quality, a lower threshold for considering a freeze-all strategy may be warranted. This aligns with emerging evidence that a freeze-all approach may be particularly beneficial in the context of premature progesterone elevation (24). In long agonist cycles, the risk-benefit calculus may differ, as the independent effect on clinical pregnancy was not statistically significant and the effect on live birth was positive after full adjustment. However, the consistently negative point estimates for clinical pregnancy across all protocols suggest that some degree of implantation impairment may be universal.

For low and normal responders—in whom the negative association with clinical pregnancy was statistically significant (OR = 0.811, *P* = 0.006 and OR = 0.900, *P* = 0.002, respectively) and who lack the compensatory reserve of multiple high-quality embryos—the clinical cost of elevated progesterone may be particularly high (25). In these patients, even a modest reduction in implantation potential may translate directly into a reduced chance of live birth. Although the association did not reach statistical significance in high responders (OR = 0.872, *P* = 0.24), the point estimate remained below unity, providing no evidence of a protective effect and suggesting that even abundant embryo availability may not fully offset the endometrial compromise.

## 5 Strengths and limitations

The principal strengths of this study include its large sample size, inclusion of multiple ovarian stimulation protocols, rigorous exclusion of key confounders, and systematic use of sequential adjustment models to isolate the independent effect of progesterone.

Several limitations should be noted. First, despite multivariable adjustment, the retrospective design precludes causal inference and may introduce residual bias. Second, the single-center design limits generalizability. Third, fresh embryo transfer criteria evolved over the 10-year study period without a uniform standard, potentially introducing temporal confounding; detailed criteria for fresh versus freeze-all decisions, yearly protocol changes (2014–2024), and excluded cycle counts are provided in **Supplementary Table 9**. Fourth, restricting the analysis to fresh transfer cycles introduces important selection bias: patients with markedly elevated trigger-day progesterone were predominantly managed with a freeze-all strategy, systematically excluding cycles at highest risk for progesterone-related implantation failure. Fifth, although embryo quality was included as a covariate, residual confounding by unmeasured embryo characteristics cannot be ruled out. Finally, certain subgroup analyses, particularly for the ultra-long protocol and the high-response stratum, may have been underpowered. Internal validation of the full prediction model incorporating the progesterone-by-protocol interaction demonstrated good calibration (corrected calibration slope ≈ 0.99) with minimal overfitting, but only moderate discriminative performance (optimism-corrected AUC: 0.622 for clinical pregnancy; 0.626 for live birth). The limited Nagelkerke R² further indicates that, while the model is well-calibrated and suitable for population-level risk stratification, its ability to guide individualized clinical decision-making remains constrained, reflecting the inherent complexity of reproductive outcomes.

## 6 Conclusion

In this large retrospective cohort, the observed association between elevated trigger-day progesterone and improved outcomes was attributable to confounding by ovarian response and embryo quality. After adjustment, elevated progesterone was independently associated with reduced clinical pregnancy rates across most contexts. A nonlinear interaction with stimulation protocol was observed: GnRH-agonist protocols demonstrated an inverted U-shaped dose–response relationship, whereas the antagonist protocol exhibited a flatter relationship with lower predicted probabilities. The association with live birth differed by protocol, with positive estimates in long agonist cycles and young patients, and negative estimates in antagonist cycles. Sensitivity analysis indicated that the advantage of agonist protocols was consistent across the progesterone range. These findings suggest that elevated progesterone may not simply reflect good ovarian response, and that both ovarian response and stimulation protocol may be relevant when evaluating its potential impact. The prediction model showed good calibration but moderate discriminative performance, indicating that additional markers may be needed for individualized risk stratification. Prospective studies incorporating endometrial receptivity markers and frozen-thawed transfer outcomes may help validate these observations and inform protocol-specific progesterone thresholds.

## Data Availability

The original contributions presented in the study are included in the article/**Supplementary Material**. Further inquiries can be directed to the corresponding authors.
