# Supplementary material for: Late follicular phase progesterone levels and in vitro fertilization and intracytoplasmic sperm injection outcomes
Source: Front Endocrinol (Lausanne). 2026 Aug 7;17:1899934. doi: 10.3389/fendo.2026.1899934 (PMC13493308; doi:10.3389/fendo.2026.1899934)
Supplement: Supplementary file 1 [file Table1.docx]

Supplement Table 1 Patient demographics, treatment protocol, pregnancy outcome and endocrine profile on the day of hCG administration (n = 24868)

| Characteristic | Value |
| --- | --- |
| **Age (years), mean ± SD** | 30.67 ± 4.55 |
| **Age group, n (%)** |  |
| < 35 years | 19,970 (80.30) |
| 35–40 years | 3,892 (15.65) |
| 40–45 years | 1,006 (4.04) |
| BMI (kg/m²), mean ± SD (range) | 23.49 ± 3.64 (16.65–60.18) |
| **Indication for IVF/ICSI, n (%)** |  |
| Tubal factor | 12,677 (50.98) |
| Sperm abnormalities | 5,977 (24.04) |
| Mixed factors | 2,784 (11.20) |
| Unexplained infertility | 1,717 (6.91) |
| Polycystic ovary syndrome | 1,132 (4.55) |
| Endometriosis | 577 (2.32) |
| **Stimulation protocol, n (%)** |  |
| Long GnRH agonist | 12,223 (49.14) |
| Ultra-long GnRH agonist | 6,347 (25.52) |
| GnRH antagonist | 6,298 (25.33) |
| **Progesterone on hCG day, n (%)** |  |
| > 1 ng/mL | 16,455 (66.17) |
| > 1.5 ng/mL | 4,834 (19.44) |
| **Fertilization method, n (%)** |  |
| Conventional IVF | 18,529 (74.51) |
| Combined IVF & ICSI | 3,270 (13.15) |
| ICSI | 3,069 (12.34) |
| **Clinical pregnancy, n (%)** | 14,706 (59.14) |
| **Miscarriage, n (%)** | 1,440 (9.79)* |
| Early miscarriage, n (%) | 945 (6.26) |
| Late miscarriage, n (%) | 495 (3.37) |
| **Live birth rate, n (%)** | 13266 (53.34) |

*Calculated per clinical pregnancy.

Supplement Table 2 Baseline Characteristics of Patients in Different Age Groups

|  | Age ＜35 y | 35y≤Age＜40y | 40y≤Age＜45y | F | p-value |
| --- | --- | --- | --- | --- | --- |
| BMI (kg/m2) | 23.39±3.72 | 23.69±3.42 | 24.20±3.46 | 38.98 | ＜0.001 |
| Basal AMH (ng/ml) | 3.76±3.02 | 2.34±2.01 | 1.51±1.45 | 195.41 | ＜0.001 |
| P on hCG day (ng/L) | 1.17±0.61 | 1.05±0.58 | 0.933±0.66 | 5.72 | ＜0.001 |
| E2 on hCG day (pg/ml) | 3265.68±1635.34 | 2516.12±1599.97 | 1817.71±1385.82 | 36.28 | ＜0.001 |
| LH on hCG day (mIU/ml) | 2.45±10.46 | 3.50±18.70 | 4.68±8.14 | 22.67 | ＜0.001 |
| Basal FSH (IU/L) | 8.23±0.49 | 8.69±4.17 | 10.18±2.24 | 0.906 | 0.404 |

BMI= Body mass index, AMH= Ant-mullerian hormone, P= progesterone, E2= Estradiol, LH= Luteinizing hormone.

Supplement Table 3 Regression analysis of clinical pregnancy rate by age group

|  | β | SE | Wald | p | OR | 95%CI |
| --- | --- | --- | --- | --- | --- | --- |
| Age group |  |  | 613.384 | <0.001 |  |  |
| Age=35-40 y | -0.514 | 0.035 | 212.774 | 0.063 | 0.598 | [0.558-0.641] |
| Age=40-45 y | -1.547 | 0.073 | 447.597 | <0.001 | 0.213 | [0.184-0.246] |

Supplement Table 4 Baseline and Ovarian Stimulation Outcomes by Progesterone (P) Level (Age <35 years)

|  | p＜1 ng/ml | p=1.0-1.25 ng/ml | p=1.25-1.5 ng/ml | p=1.5-1.75ng/ml | p=1.75-2.0ng/ml | p=2.0-2.25 ng/ml | p=2.25-2.5 ng/ml | p＞2.5 ng/ml | F | p-value |
| --- | --- | --- | --- | --- | --- | --- | --- | --- | --- | --- |
| Age | 29.17±3.12 | 28.99±3.12 | 28.94±3.06 | 28.80±3.22 | 28.86±3.08 | 28.79±3.07 | 28.48±3.49 | 29.21±3.06 | 378.41 | ＜0.001 |
| BMI | 23.97±3.81 | 23.21±3.58 | 23.02±3.57 | 22.89±3.58 | 22.70±3.63 | 22.33±3.39 | 22.45±3.63 | 22.41±3.50 | 65.243 | ＜0.001 |
| AMH | 3.38±2.88 | 3.69±3.04 | 3.86±2.83 | 4.10±3.18 | 4.42±3.19 | 4.85±3.59 | 4.55±2.38 | 5.28±3.71 | 21.805 | ＜0.001 |
| LH on hcg day | 2.57±13.83 | 2.41±4.66 | 2.31±3.06 | 2.27±2.21 | 2.39±14.27 | 2.34±2.49 | 2.43±3.51 | 2.37±3.02 | 0.83 | 0.563 |
| E2 on hcg day | 2673.40±1684.83 | 3392.99±1410.25 | 3641.69±1611.42 | 3815.33±1323.44 | 4071.58±1286.20 | 3971.68±1325.57 | 4133.45±1230.66 | 4174.63±1490.46 | 410.83 | ＜0.001 |
| Gn days | 10.47±2.59 | 10.55±2.34 | 10.56±2.12 | 10.63±2.09 | 10.78±2.17 | 10.58±2.0 | 10.92±2.0a | 10.79±1.94 | 7.181 | ＜0.001 |
| Total Gn | 2502.19±1211.73 | 2519.84±2505.73 | 2470.70±932.15 | 2480.84±856.39 | 2506.13±837.71 | 2513.32±816.11 | 2561.09±810.25 | 2495.77±864.14 | 0.415 | 0.894 |
| Number of retrieved oocytes | 9.40±4.97 | 10.90±5.11 | 11.45±5.08 | 11.69±5.17 | 12.33±5.05 | 12.64±5.12 | 13.41±5.62 | 12.13±6.23 | 126.654 | ＜0.001 |
| Number of MII oocytes | 4.27±4.61 | 4.89±5.13 | 5.15±5.33 | 4.91±5.35 | 5.22±5.65 | 3.59±5.62 | 3.14±5.39 | 2.46±4.84 | 32.564 | ＜0.001 |
| Number of 2PN | 5.01±3.98 | 5.50±4.41 | 5.55±4.55 | 5.34±4.61 | 5.19±4.88 | 5.47±5.11 | 5.46±5.12 | 3.79±4.95 | 13.053 | ＜0.001 |
| Number of high-quality embryo | 1.32±2.02 | 1.42±2.21 | 1.40±2.14 | 1.30±2.14 | 1.07±1.89 | 1.44±2.22 | 1.58±2.37 | 1.11±2.19 | 8.791 | ＜0.001 |

BMI= Body mass index, AMH= Ant-mullerian hormone, P= progesterone, E2= Estradiol, LH= Luteinizing hormone, Gn= Gonadotropin, MII=Metaphase II, 2PN=fertilized oocytes

Supplementary Table 5. Multivariate regression analysis of clinical pregnancy rate

|  | β | SE | Wald | p | OR | 95%CI |
| --- | --- | --- | --- | --- | --- | --- |
| **Clinical Pregnancy Rate** |  |  |  |  |  |  |
| Age (＜35 years vs. ≥35 years) | -0.429 | 0.039 | 124.042 | <0.001 | 0.651 | [0.604,0.702] |
| BMI (kg/m2) | -0.012 | 0.004 | 7.717 | 0.005 | 0.988 | [0.980,0.997] |
| AMH | -0.043 | 0.010 | 17.594 | <0.001 | 0.958 | [0.939,0.977] |
| FSH | 0.000 | 0.000 | 0.024 | 0.878 | 1.000 | [1.000,1.000] |
| Protocols (long GnRH-a vs. GnRH-ant) | -0.527 | 0.037 | 198.640 | <0.001 | 0.590 | [0.548,0.635] |
| p on hCG day(ng/ml) | 0.095 | 0.031 | 9.328 | 0.002 | 1.099 | [1.035,1.168] |
| E2 on hCG day | 0.000 | 0.000 | 4.703 | 0.030 | 1.000 | [1.000,1.000] |
| LH on hCG day(ng/ml) | 0.001 | 0.001 | 0.485 | 0.486 | 1.001 | [0.998,1.004] |
| Fertilization method (IVF vs. ISCI) | 0.077 | 0.039 | 5.202 | 0.023 | 1.080 | [1.011,1.155] |
| **Live Brith Rate** |  |  |  |  |  |  |
| Age (＜35 years vs. ≥35 years) | -0.581 | 0.041 | 198.977 | <0.001 | 0.559 | [0.516,0.606] |
| BMI (kg/m2) | -0.003 | 0.004 | 0.539 | 0.463 | 0.997 | [0.989,1.005] |
| AMH | -0.036 | 0.010 | 12.530 | <0.001 | 0.964 | [0.945,0.984] |
| FSH | 0.000 | 0.000 | 0.056 | 0.814 | 1.000 | [1.000,1.000] |
| Protocols (long GnRH-a vs. GnRH-ant) | -0.654 | 0.040 | 271.330 | <0.001 | 0.520 | [0.481,0.562] |
| p on hCG day(ng/ml) | 0.034 | 0.031 | 1.161 | 0.281 | 1.034 | [0.973,1.100] |
| E2 on hCG day | 0.000 | 0.000 | 9.840 | 0.002 | 1.000 | [1.000,1.000] |
| LH on hCG day(ng/ml) | 0.001 | 0.002 | 0.242 | 0.623 | 1.001 | [0.998,1.004] |
| Fertilization method (IVF vs. ISCI) | 0.110 | 0.034 | 10.182 | 0.001 | 1.116 | [1.043,1.194] |

Abbreviation= BMI, body mass index; AMH= anti-Müllerian hormone; GnRH-a = gonadotropin-releasing hormone agonist; GnRH-ant = gonadotropin-releasing hormone antagonist; p= progesterone; E2= estradiol; hCG= human chorionic gonadotropin; LH= luteinizing hormone

Supplement Table 6 Baseline Characteristics by Ovarian Stimulation Protocol

|  | Long GnRHa | Ultra-long GnRHa | GnRHant | F | p |
| --- | --- | --- | --- | --- | --- |
| Age | 30.12±4.22 | 29.79±4.09 | 32.65±5.07 | 855.891 | ＜0.001 |
| Body mass index | 23.02±3.44 | 24.11±3.90 | 23.79±3.73 | 207.589 | ＜0.001 |
| Basal AMH (pg/ml) | 3.26±2.21 | 4.65±3.30 | 2.78±2.95 | 374.672 | ＜0.001 |
| P on hCG day (ng/L) | 1.16±0.54 | 1.14±0.61 | 1.10±0.64 | 25.002 | ＜0.001 |
| E2 on hCG day (pg/ml) | 3553.68±1623.93 | 3256.90±1472.25 | 2347.36±1600.66 | 1222.409 | ＜0.001 |
| LH on hCG day (mIU/ml) | 2.10±4.16 | 1.53±15.42 | 4.20±12.64 | 119.154 | ＜0.001 |
| Number of retrieved oocytes | 10.96±4.99 | 11.33±5.0 | 7.42±4.74 | 1069.351 | ＜0.001 |

AMH= Ant-mullerian hormone, P= progesterone, E2= Estradiol, LH= Luteinizing hormone

Supplementary Table 7. Impact of progesterone levels on clinical pregnancy rate (CPR), miscarriage rate (MR), and live birth rate (LBR) among women undergoing different ovulation induction protocols

|  | p＜1 ng/ml | p=1.0-1.25 ng/ml | p=1.25-1.5 ng/ml | p=1.5-1.75ng/ml | p=1.75-2.0ng/ml | p=2.0-2.25 ng/ml | p=2.25-2.5 ng/ml | p＞2.5 ng/ml | χ² | p-value |
| --- | --- | --- | --- | --- | --- | --- | --- | --- | --- | --- |
| Clinical pregnancy rate | |  |  |  |  |  |  |  |  |  |
| Long GnRH-a | 3061/5115(59.8%) | 1513/2470(61.3%) | 1201/1860(64.6%) | 698/1109(62.9%) | 723/1173(61.6%) | 119/209(56.9%) | 58/118(49.2%) | 115/169(68.0%) | 26.514 | ＜0.001 |
| Ultra-long GnRH-a | 1883/2920(64.5%) | 714/1066(67.0%) | 559/844(66.2%) | 391/542(72.1%) | 541/769(70.4%) | 57/81(70.4%) | 32/45(71.1%) | 62/80(77.5%) | 23.496 | 0.001 |
| GnRH-ant | 1339/3048(43.9%) | 495/1062(46.6%) | 392/846(46.3%) | 289/540(53.5%) | 343/634(54.1%) | 49/78(62.8%) | 20/27(74.1%) | 52/63(82.5%) | 81.231 | ＜0.001 |
| Miscarriage rate |  |  |  |  |  |  |  |  |  |  |
| Long GnRH-a | 307/3061(10.0%) | 145/1513(9.6%) | 101/1201(8.4%) | 52/698(7.4%) | 41/723(5.7%) | 5/119(4.2%) | 3/58(5.2%) | 14/115(12.2%) | 22.8541 | 0.002 |
| Ultra-long GnRH-a | 186/1883(9.9%) | 72/714(10.1%) | 49/559(8.8%) | 25/391(6.4%) | 32/541(5.9%) | 3/57(5.3%) | 2/32(6.3%) | 0/62(0.0%) | 19.902 | 0.006 |
| GnRH-ant | 217/1339(16.2%) | 60/495(12.1%) | 53/392(13.5%) | 24/289(8.3%) | 25/343(7.3%) | 2/49(4.1%) | 1/20(5.0%) | 1/52(1.9%) | 38.656 | ＜0.001 |
| Early miscarriage rate | |  |  |  |  |  |  |  |  |  |
| Long GnRH-a | 173/3061(5.7%) | 92/1513(6.1%) | 71/1201(5.9%) | 34/698(4.9%) | 25/723(3.5%) | 2/119(1.7%) | 2/58(3.4%) | 6/115(5.2%) | 11.732 | 0.110 |
| Ultra-long GnRH-a | 110/1883(5.8%) | 52/714(7.3%) | 30/559(5.4%) | 17/391(4.3%) | 17/541(3.1%) | 2/57(3.5%) | 0/32(0.0%) | 0/62(0.0%) | 17.749 | 0.013 |
| GnRH-ant | 168/1339(12.5%) | 49/495(9.9%) | 38/392(9.7%) | 16/289(5.5%) | 17/343(5.0%) | 2/49(4.1%) | 1/20(5.0%) | 1/52(1.9%) | 32.464 | ＜0.001 |
| Late miscarriage rate | |  |  |  |  |  |  |  |  |  |
| Long GnRH-a | 134/3061(4.4%) | 53/1513(3.5%) | 30/1201(2.5%) | 18/698(2.6%) | 16/723(2.2%) | 3/119(2.5%) | 1/58(1.7%) | 8/115(7.0%) | 20.725 | 0.004 |
| Ultra-long GnRH-a | 76/1883(4.0%) | 20/714(3.2%) | 19/559(3.4%) | 8/391(2.0%) | 15/541(2.8%) | 1/57(1.8%) | 2/32(6.3%) | 0/62(0.0%) | 9.503 | 0.219 |
| GnRH-ant | 49/1339(3.7%) | 11/495(2.2%) | 15/392(3.8%) | 8/289(2.8%) | 8/343(2.3%) | 0/49(0.0%) | 0/20(0.0%) | 0/52(0.0%) | 8.098 | 0.324 |
| Live birth delivery rate | |  |  |  |  |  |  |  |  |  |
| Long GnRH-a | 2597/5115(50.8%) | 1314/2470(53.2%) | 1064/1860(57.2%) | 623/1109(56.2%) | 668/1173(56.9%) | 110/209(52.6%) | 54/118(45.8%) | 99/169(58.6%) | 30.060 | ＜0.001 |
| Ultra-long GnRH-a | 1637/2920(56.1%) | 633/1066(59.4%) | 497/844(58.9%) | 364/542(67.2%) | 505/769(65.7%) | 52/81(64.2%) | 29/45(64.4%) | 62/80(77.5%) | 51.771 | ＜0.001 |
| GnRH-ant | 986/3048(32.3%) | 391/1062(36.8%) | 313/846(37.0%) | 249/540(46.1%) | 308/634(48.6%) | 45/78(58.4%) | 19/27(70.4%) | 51/63(81.0%) | 161.864 | ＜0.001 |

GnRH-a = gonadotropin-releasing hormone agonist; GnRH-ant = gonadotropin-releasing hormone antagonist.

Supplement Table 8 Comparisons of Progesterone Subgroups in all patients

|  | p＜1 ng/ml | p=1.0-1.25 ng/ml | p=1.25-1.5 ng/ml | p=1.5-1.75ng/ml | p=1.75-2.0ng/ml | p=2.0-2.25 ng/ml | p=2.25-2.5 ng/ml | p＞2.5 ng/ml | F/χ² | p-value |
| --- | --- | --- | --- | --- | --- | --- | --- | --- | --- | --- |
| Age | 31.21±4.78 | 30.49±4.47 | 30.32±4.30 | 30.12±4.37 | 30.02±4.19 | 29.72±4.02 | 29.73±4.19 | 29.84±3.95 | 45.124 | ＜0.001 |
| BMI | 24.05±3.74 | 23.29±3.55 | 23.12±3.53 | 22.96±3.55 | 22.80±3.58 | 22.26±3.28 | 22.20±3.42 | 22.57±3.61 | 75.588 | ＜0.001 |
| AMH | 2.97±2.69 | 3.40±2.88 | 3.61±2.79 | 3.83±3.06 | 4.19±3.09 | 4.80±3.76 | 4.40±2.32 | 5.27±3.64 | 47.385 | ＜0.001 |
| E2 on hCG day | 2558.22±1679.25 | 3333.22±1437.40 | 3593.02±1600.17 | 3805.16±1329.48 | 4066.12±1286.15 | 4100.16±1256.03 | 4137.00±1276.26 | 4323.89±1399.45 | 523.684 | ＜0.001 |
| Number of retrieved oocytes | 8.92±4.95 | 10.60±5.09 | 11.19±5.08 | 11.40±5.09 | 12.16±5.08 | 12.71±5.14 | 12.95±5.34 | 12.55±5.98 | 177.299 | ＜0.001 |
| Clinical pregnancy rate | 6823/11082(61.6%) | 2722/4598(59.2%) | 2152/3550(60.6%) | 1378/2191(62.9%) | 1607/2576(62.4%) | 225/368(61.1%) | 110/190(57.9%) | 229/312(73.4%) | 33.382 | ＜0.001 |
| Miscarriage rate | 666/6283(10.6%) | 304/2722(11.2%) | 173/2152(8.0%) | 116/1378(8.4%) | 138/2576(5.4%) | 23/225(10.2%) | 9/110(8.2%) | 11/229(4.8%) | 83.208 | ＜0.001 |
| Live birth rate | 5220/11082(47.1%) | 2338/4598(50.8%) | 1874/3550(52.8%) | 1236/2191(56.4%) | 1481/2576(57.5%) | 207/368(56.3%) | 102/190(53.7%) | 212/312(67.9%) | 181.493 | ＜0.001 |

BMI= Body mass index, AMH= Ant-mullerian hormone, P= progesterone, E2= Estradiol.

Supplement Table 9: Evolution of Guidelines and Consensus on Fresh vs. Freeze-All Strategy Decision-Making in IVF (2014–2024)

| Time Period | Key Guidelines/Consensus/Milestone Studies | Core Recommendations on Progesterone (P) Threshold and Freeze-All Strategy | Impact on Clinical Practice |
| --- | --- | --- | --- |
| Pre-2014 & Early Phase | Multiple retrospective studies | It was proposed that elevated progesterone on the day of hCG administration (common cut-off values: >1.5, >1.75, or >2.0 ng/mL) might impair endometrial receptivity, thereby reducing the live birth rate in fresh cycles. A unified international guideline was lacking. | Centers adopted empirical thresholds, leading to considerable variation. Fresh transfer was mostly cancelled for patients exceeding the cut-off. |
| 2015–2018 | 2016: Chen ZJ's team's PCOS freeze-all study (NEJM) 2017: Chinese Expert Consensus on the Impact of High Progesterone in the Follicular Phase on IVF/ICSI Outcomes | The 2017 Chinese consensus explicitly recommended: When progesterone on the day of hCG administration is >1.5 ng/mL, endometrial receptivity is deemed compromised. It is advisable to cancel fresh embryo transfer and implement a freeze-all strategy. | The freeze-all concept gained widespread acceptance. A large number of domestic centers uniformly and strictly adopted 1.5 ng/mL as the progesterone threshold for freeze-all. |
| 2019–2021 | 2020: ESHRE Guideline on Ovarian Stimulation | For the first time, an international authoritative guideline explicitly recommended: For conventional IVF cycles, if progesterone on the day of hCG administration is >1.5 ng/mL, deferring fresh transfer and adopting a freeze-all strategy should be considered. | 1.5 ng/mL became an internationally recognized reference threshold and was adopted as a routine clinical standard by many centers. |
| 2022–2024 | Recent large-scale retrospective studies and subgroup analyses | Challenged the absolute threshold, proposing: • When progesterone is only mildly elevated (e.g., 1.5–2.0 ng/mL) and embryo quality is high, the live birth rate from fresh transfer may be comparable to that from frozen-thawed transfer. • It is recommended to make individualized decisions integrating factors such as age and embryo grade, rather than applying a "one-size-fits-all" freeze-all approach to all patients with P >1.5 ng/mL. | Clinical practice has once again become divergent: Some centers maintain the strict 1.5 ng/mL standard; others have relaxed the threshold to 2.0 ng/mL or adopted a comprehensive assessment based on embryo quality. |

多因素回归分析

|  | β | SE | Wald | p | OR | 95%CI |
| --- | --- | --- | --- | --- | --- | --- |
| Age | -0.494 | 0.039 | 164.358 | <0.001 | 0.610 | [0.566-0.658] |
| BMI (kg/m2) | 0.023 | 0.006 | 16.073 | <0.001 | 1.023 | [1.012-1.034] |
| Basal AMH (ng/ml) | 0.101 | 0.10 | 112.320 | <0.001 | 1.107 | [1.086-1.127] |
| P on hCG day (ng/L) | 0.089 | 0.041 | 4.708 | 0.030 | 1.093 | [1.009-1.185] |
| E2 on hCG day (pg/ml) | <0.001 | <0.001 | 49.267 | <0.001 | 1.000 | [1.000-1.000] |
| LH on hCG day (mIU/ml) | -0.003 | 0.004 | 0.610 | 0.435 | 0.997 | [0.989-1.005] |
| Protocols | -0.213 | 0.025 | 73.463 | <0.001 | 0.808 | [0.770-0.848] |

**Hosmer-Lemeshow test**：χ²(8) = 8.465，p = 0.389

多因素回归分析（分组）

|  | β | SE | Wald | p | OR | 95%CI |
| --- | --- | --- | --- | --- | --- | --- |
| Age (＜35 years vs. ≥35 years) | -0.535 | 0.049 | 120.545 | <0.001 | 0.568 | [0.533-0.645] |
| BMI (＜28 kg/m^2^ vs. ≥28kg/m2) | -0.069 | 0.061 | 1.274 | 0.295 | 0.934 | [0.829-1.052] |
| AMH (>3.5 ng/ml vs. ≤3.5 ng/ml) | 0.546 | 0.047 | 133.952 | <0.001 | 1.727 | [1.574-1.895] |
| Protocols (long GnRHa vs. GnRHant) | 0.476 | 0.043 | 120.952 | <0.001 | 1.610 | [1.479-1.752] |
| p on hCG day(ng/ml) | 0.144 | 0.040 | 12.747 | <0.001 | 1.155 | [1.067-1.250] |
| E2 on hCG day(≥3000 pg/ml vs.＜3000 pg/ml) | 0.291 | 0.047 | 37.940 | <0.001 | 1.338 | [1.220-1.468] |
| LH on hCG day(ng/ml) | -0.003 | 0.004 | 0.605 | 0.437 | 0.997 | [0.990-1.004] |

**Hosmer-Lemeshow test**：χ²(8) = 8.465，p = 0.389
